# A Benchmark of Genetic Variant Calling Pipelines Using Metagenomic Short-Read Sequencing

**DOI:** 10.3389/fgene.2021.648229

**Published:** 2021-05-10

**Authors:** Sergio Andreu-Sánchez, Lianmin Chen, Daoming Wang, Hannah E. Augustijn, Alexandra Zhernakova, Jingyuan Fu

**Affiliations:** ^1^Department of Genetics, University of Groningen and University Medical Center Groningen, Groningen, Netherlands; ^2^Department of Pediatrics, University of Groningen and University Medical Center Groningen, Groningen, Netherlands

**Keywords:** metagenomics, shotgun sequencing, short-reads, variant-calling, benchmark

## Abstract

Microbes live in complex communities that are of major importance for environmental ecology, public health, and animal physiology and pathology. Short-read metagenomic shotgun sequencing is currently the state-of-the-art technique for exploring these communities. With the aid of metagenomics, our understanding of the microbiome is moving from composition toward functionality, even down to the genetic variant level. While the exploration of single-nucleotide variation in a genome is a standard procedure in genomics, and many sophisticated tools exist to perform this task, identification of genetic variation in metagenomes remains challenging. Major factors that hamper the widespread application of variant-calling analysis include low-depth sequencing of individual genomes (which is especially significant for the microorganisms present in low abundance), the existence of large genomic variation even within the same species, the absence of comprehensive reference genomes, and the noise introduced by next-generation sequencing errors. Some bioinformatics tools, such as metaSNV or InStrain, have been created to identify genetic variants in metagenomes, but the performance of these tools has not been systematically assessed or compared with the variant callers commonly used on single or pooled genomes. In this study, we benchmark seven bioinformatic tools for genetic variant calling in metagenomics data and assess their performance. To do so, we simulated metagenomic reads to mimic human microbial composition, sequencing errors, and genetic variability. We also simulated different conditions, including low and high depth of coverage and unique or multiple strains per species. Our analysis of the simulated data shows that probabilistic method-based tools such as HaplotypeCaller and Mutect2 from the GATK toolset show the best performance. By applying these tools to longitudinal gut microbiome data from the Human Microbiome Project, we show that the genetic similarity between longitudinal samples from the same individuals is significantly greater than the similarity between samples from different individuals. Our benchmark shows that probabilistic tools can be used to call metagenomes, and we recommend the use of GATK’s tools as reliable variant callers for metagenomic samples.

## Introduction

Short-read metagenomic sequencing is the technique most widely used to explore the natural habitat of millions of bacteria. In comparison with 16S rRNA sequencing, shotgun metagenomic sequencing (MGS) provides sequence information of the whole genomes, which can be used to identify different genes present in an individual bacterium and enables the examination of other genomic features such as gene synteny or genetic variation. In recent years, MGS datasets have been generated to explore the composition of the gut microbiome in a number of large human cohorts (Human Microbiome Project Consortium, 2012; Zhernakova et al., 2016; Lloyd-Price et al., 2017; Gacesa et al., 2020; Salosensaari et al., 2020). Large inter-individual variation in gut microbial composition has been widely observed, and variations in composition have been linked to lifestyle, host genetics, health, and disease. However, most of these associations reflect variations in microbial diversity and bacterial abundance, and our understanding of the genetic variations within gut bacteria is still limited.

Enthusiasm is now rising for techniques that can assess the genetic variation in the gut microbiome, which would allow us to pinpoint the putative causal bacterial genes underlying the observed associations and thereby generate testable hypotheses for mechanistic research. Single-nucleotide variation (SNV) refers to a one-nucleotide difference in a homologous region of at least two organisms. SNVs are of major importance for understanding the role of genetics in evolution, disease, phenotypes, or population genetics dynamics. The first major attempt to explore the bacterial genetic landscape revealed 10.3 million SNVs as well as many other types of genetic variants in 252 fecal samples (Schloissnig et al., 2013). However, there have been few efforts to assess the inter-individual differences in bacterial genetic profiles.

Despite its potential, SNV calling in a metagenome remains challenging. Many factors hamper the widespread application of variant-calling analysis, including the low-depth sequencing of individual genomes (which is especially significant for microorganisms present in lower abundance), large genomic variation (even within the same species), the absence of comprehensive reference genomes, and the noise introduced by next-generation sequencing errors. A plethora of different software have been produced to separate SNVs from sequencing errors after genomic mapping to a known reference. However, most tools require deeply sequenced single genomes with a known ploidy and, in all cases, mapping to a homologous region for proper function. Metagenomes also contain an unknown number of haploid organisms. Additionally, the identification of homologous regions is complicated by the presence of other bacteria that share the same evolutionary history and by possible horizontal gene-transfer events.

At present, there are several tools that have been developed specifically for metagenomic variant calling, such as MetaSNV (Costea et al., 2017) and InStrain (Olm et al., 2021). However, other variant callers have also been designed to be ploidy naïve or to address complications like an unknown number of pooled samples, including VarScan2 (Koboldt et al., 2012), freebayes (Garrison and Marth, 2012), and GATK’s Mutect2 (DePristo et al., 2011). Other widely used variant-calling tools in the world of genomics include BCFtools and GATK’s HaplotypeCaller (DePristo et al., 2011). All-in-all, these tools fall into two categories: *probabilistic tools* that calculate probabilities for a genotype given the read depth and quality of the base pairs (e.g., BCFtools, Mutect2, HaplotypeCaller, and freebayes) and *non-probabilistic* tools that call variants that pass specific thresholds such as minimal read depth or supporting reads (Table 1). While variant-calling benchmarks have been carried out in the context of bacterial variation (Yoshimura et al., 2019; Bush et al., 2020), currently, there is no benchmark on the metagenomic realm, where more complex issues exist.

**TABLE 1:** Summary of tools benchmarked and used for different analyses.

Tool name	Probabilistic	Pool population	Joint calling	Minimal coverage	ROC curve	Real data
BCFtools	Yes	No	Yes	No	Yes	No
freebayes	Yes	Yes	Yes	No	Yes	No
HaplotypeCaller	Yes	No	Yes	No	Yes	Yes
Mutect2	Yes	Yes	Yes	No	No	Yes
VarScan2	No	Yes	No	8	No	No
metaSNV	No	Yes	Yes	4	No	No
InStrain	No	Yes	No	5	No	No

We therefore aimed to benchmark different variant-calling tools in the context of metagenomes. We simulated complex metagenomic communities based on the 45 most abundant and prevalent gut microbial species across populations and disease groups (Gupta et al., 2020), which correspond, on average, to 74% of the human gut metagenome composition. We then applied seven tools to this simulated data and compared their performance under different scenarios. We further applied the tools that showed best performance on the simulated data, Mutect2 and HaplotypeCaller, to longitudinal, metagenomic-sequenced data from the Human Microbiome Project (HMP) (Schloissnig et al., 2013). This revealed the high individual specificity of microbial genetic variants, which allows them to be used to distinguish samples from the same individual taken at different times, with more power than bacterial taxonomic abundance.

## Materials and Methods

### Bacterial Species Selection and Reference Genome Download

To determine which references would be used for variant calling, we selected the 48 most abundant (mean relative abundance > 0.5%) and prevalent (presence rate > 20%) bacterial species from an integrated dataset of 4,347 publicly available human stool metagenomes, which were pooled across multiple studies encompassing various disease states (Gupta et al., 2020). These 48 species accounted for a mean total abundance of 81% (Supplementary Table 1), indicating that they capture a substantial proportion of human gut microbial composition. From these, three unclassified species were removed because no clear reference genome could be selected. The remaining 45 species accounted for 74% of mean abundance. To reach a 100% composition, we included one extra species (*Streptococcus australis*) with a dummy high abundance of 26%. We then used InSilicoSeq’s (Gourlé et al., 2019) Download_ncbi script to query GenBank for the assemblies of the selected species using Biopython’s entrez (Cock et al., 2009) Python package. When multiple assemblies were found for a given bacterial taxon, a reference genome was randomly selected from among the available assemblies. The reference used and the quality statistics are presented in Supplementary Table 2. Quality statistics measured include number of contigs; total length of the genome; minimum and maximum contig length; N50, N75, and N90 (shortest contig length needed to cover 50, 75, and 90% of the genome, respectively); and auN (area under the curve of all possible Nx metrics).

### Synthetic Read Generation

We considered two different scenarios: a *uni-strain scenario* in which only one dominant strain exists per species and a *multi-strain scenario* where two dominant strains exist per species. We generated two sets of synthetic variants, considered true positives (TP), by randomly changing 1% of the total nucleotides in each of the reference genomes (including the dummy taxa). The choice of this SNV rate was based on a previous estimate that found that the SNV diversity of most intestinal species was around 1% (range 0.018–3.9%) (Truong et al., 2017). The first dataset was used for the uni-strain scenario. The second dataset was then combined with the first, and the combined set used as the multi-strain scenario. Additionally, we repeated the simulation with 4% variation to reflect highly divergent strains and assess whether tools performance differed for highly divergent species.

Using the mutated reference genomes, we ran InSilicoSeq (iss generate) (Gourlé et al., 2019) on the known bacterial taxonomy table (–abundance_file) to generate a simulated set of ∼15 million Hiseq paired-end reads, a sequencing depth similar to other metagenomic datasets (Zhernakova et al., 2016; Byrd et al., 2021). InSilicoSeq simulates reads using an error model based on Illumina’s Hiseq technology. We can estimate the expected genome coverage by adjusting the Lander–Waterman estimation method for computing coverage by the abundance of the taxon (Lander and Waterman, 1988). Using the reference genomic length, simulated abundance, read length (126 bp), and number of reads, we estimated the expected coverage for each of the microbial species using Equation (1).

$$E\,x\,p\,e\,c\,t\,e\,d\,c\,o\,v\,e\,r\,a\,g\,e_{i}=\frac{R\,e\,a\,d\,s\times 126\times A\,b\,u\,n\,d\,a\,n\,c\,e_{i}}{G\,e\,n\,o\,m\,e\,l\,e\,n\,g\,t\,h_{i}}$$

**Equation 1.** Expected coverage of a given species i. Reads is a constant per simulation indicating the number of simulated reads. Abundance indicates the relative abundance of a species (0–1). Genome length is the total number of base pairs in the reference genome of a species i.

### Read Trimming

The simulated dataset was trimmed following a typical metagenomics pipeline. We removed low-quality reads from the raw metagenomic sequencing data using KneadData (version 0.7.4). KneadData can also remove host genome-contaminated reads, which should not exist in the simulated scenario, but is necessary in real-life human-derived microbiome projects. KneadData uses Bowtie2 (version 2.3.4.3) (Langmead and Salzberg, 2012) and Trimmomatic (version 0.39) (Bolger et al., 2014). In brief, the data-cleaning procedure includes two main steps: (1) filtering out of the human genome-contaminated reads by aligning raw reads to the human reference genome (GRCh37/hg19) and (2) removal of adaptor sequences using Trimmomatic (default trimming: SLIDINGWINDOW:4:20 MINLEN:70).

### Genome Mapping

We used the standard setting (*–sensitive* mode) of Bowtie2 (version 2.3.4.3) (Langmead and Salzberg, 2012) to map the simulated metagenomic reads to the unmutated original reference genomes (the reference genomes were mapped one at a time), using default options. Reads were sorted using SAMtools (version 1.9) (Li et al., 2009), and duplicates were marked and removed by running the MarkDuplicates module (version 2.18.26-SNAPSHOT) (*REMOVE_DUPLICATES* = *True*) of Picard. We cleaned the resulting BAM files using the CleanSam module (version 2.18.26-SNAPSHOT) of Picard.

### Redundancy of Genome Assessment

To compute the similarity between the 46 chosen genomes, we used Mash (Ondov et al., 2016) with a k-mer size of 17 and a sketch size of 10,000. In addition, we estimated the proportion of multi-mapping reads in a combined reference of all 46 species. With this, we aimed to characterize how much genome homology would impact read assignment. We therefore extracted the information regarding the number of concordant reads (i.e., both pairs mapping meaningfully), concordant reads with multiple equally good mapping positions, pairs that mapped non-concordantly, unpaired reads mapped uniquely, and unpaired reads mapping to multiple positions. We estimated the number of multi-mappers by summing both paired mapped and unpaired mapped equally well mapping reads (Equation 2).

$$M\,u\,l\,t\,i\,\_\,m\,a\,p\,p\,i\,n\,g\,r\,a\,t\,e=\frac{2\times \left(M\,u\,l\,t\,i\,\_\,m\,a\,p\,p\,e\,d\,p\,a\,i\,r\,s\right)+M\,u\,l\,t\,i\,\_\,m\,a\,p\,p\,e\,d\,u\,n\,p\,a\,i\,r\,e\,d\,r\,e\,a\,d\,s}{2\times P\,a\,i\,r\,e\,d\,r\,e\,a\,d\,n\,u\,m\,b\,e\,r}$$

**Equation 2**. Multi-mapping rate. The number of multi-mapped reads include two times the number of multi-mapped pairs and the unpaired reads that were mapped to multiple positions.

In addition, if we consider non-concordant pairs as reads mapping to an incorrect position (since there are no structural variations in the reference), we can get a second estimate of the reads mapping to positions other than their origin (Equation 3).

$$I\,n\,c\,o\,r\,r\,e\,c\,t\,o\,r\,m\,u\,l\,t\,i\,m\,a\,p\,p\,i\,n\,g\,r\,a\,t\,e=\frac{N\,o\,n\,\_\,c\,o\,n\,c\,o\,r\,d\,a\,n\,t\,p\,a\,i\,r\,s+M\,u\,l\,t\,i\,\_\,m\,a\,p\,p\,i\,n\,g\,r\,e\,a\,d\,s}{2\times P\,a\,i\,r\,e\,d\,r\,e\,a\,d\,n\,u\,m\,b\,e\,r}$$

**Equation 3**. Incorrect or multi-mapping rate. The number of multi-mapped reads include two times the number of multi-mapped pairs and the unpaired reads that were mapped to multiple positions. Non-concordant pairs refer to pairs where both reads mapped to a single position but do not follow the expected read orientation.

### Variant Calling

Using the cleaned BAM files and the reference (not-mutated) genomes, we performed variant calling using the following tools and specifications.

#### BCFtools

BCFtools variant calling is based on BCFtool’s mpileup output. For each bacterial alignment, we used mpileup (default options) and BCFtools call. Ploidy was set to 1, and we used the multi-allelic calling algorithm (-m). The BCFtools algorithm does not consider a population of pooled samples, and as we run it on a sample-by-sample basis, it only assesses two possible genotypes: reference or alternative. It is also worth noting that in order to calculate likelihoods, BCFtools uses a prior based on the human effective population size (theta) of 0.001^1^.

#### Freebayes

freebayes is a haplotype-based variant caller (Garrison and Marth, 2012). This means that instead of calling variants position-by-position based on an aligned read, it checks the whole haplotype of the read independently of the precise alignment positions. This solves the issue of multiple ambiguous alignment possibilities between the read and the homologous genomic region. We set ploidy to 1. As we have an unknown number of pooled samples (bacteria) that might align with a homologous region, we set the parameter –pooled-continuous. The joint-calling options –min-alternate-count and –min-alternate-fraction were set to 2 and 0, respectively.

#### HaplotypeCaller

GATK’s assembly-based variant caller HaplotypeCaller (DePristo et al., 2011) is able to handle non-diploid organisms as well as pooled experiment data. We therefore applied HaplotypeCaller with default settings, with the exception of setting ploidy to 1. Haplotypes are called by HaplotypeCaller via local re-assembly of regions of a potential variant site, from which a pair-HMM alignment of reads to haplotypes is generated. In the final step, the algorithm determines the likelihoods of the genotypes and reports the most likely genotype at each site^2^.

#### Mutect2

GATK’s Mutect2 (DePristo et al., 2011) uses a similar approach to HaplotypeCaller in calling variants, including active region-based identification, assembly-based haplotype reconstruction, and pair-HMM alignment of reads to haplotypes. However, whereas HaplotypeCaller is designed to call germline variants, Mutect2 is designed to call somatic variants. Mutect2 therefore includes somatic-specific genotyping and filtering steps. It is designed to have a high specificity but cannot calculate reference confidence and define ploidy. We employed Mutect2 in tumor-only mode with default settings but including “–af-of-alleles-not-in-resource 0.33,” as recommended when using a non-human organism as input^3^.

#### VarScan2

VarScan2 employs a heuristic approach to call variance that relies on parameter thresholds to determine variants (Koboldt et al., 2012). Given a SAMtools mpileup-formatted alignments file, VarScan2 first performs a read-filtering step that discards any reads that align to multiple locations or do not comply with the quality criteria. VarScan2 then screens the alignments on a per-read basis to detect sequence variance and merges variants detected in multiple reads into unique SNPs and indels. Only variants meeting user-defined parameter thresholds are reported. Here, we applied the VarScan2 mpileup2snp algorithm using default settings, including minimum read depth of 8, base quality of 15, supporting reads of 2, allele frequency of 0.01, and a Fisher’s exact test *p*-value below 0.99.

#### MetaSNV

MetaSNV was specifically designed for metagenomic datasets and can handle large multi-species references (Costea et al., 2017). We applied the default parameters of metaSNV for SNV calling. metaSNV determines the existence of a candidate variant on a per-nucleotide basis, building upon the mpileup tool in the Samtools suite (Li et al., 2009). All reads from all samples that align to a given position are considered together. If at least four variant-containing reads cover a position (across all samples), it is considered a potential SNV. Variants are split into two classes: population and individual variants. Population variants are non-reference nucleotides observed in > 1% of all reads combined across all samples. Individual variants are those that fall below the 1% population frequency threshold but are confidently observed in at least one sample (at least four reads containing the variant). If multiple different non-reference nucleotides are observed, all are reported independently.

#### InStrain

InStrain (Olm et al., 2021) was devised to detect SNVs and profile intra-population genetic diversity based on metagenomic short-read alignment. InStrain first performs read filtering to remove read pairs that do not meet the quality criteria. Then, for each position with multiple aligned reads supported in the reference genome, both biallelic and multiallelic SNVs are identified by detecting bases that are different from the reference genome at the same position. The frequencies of SNVs are also counted. Additionally, if the gene annotation for the reference genome is provided, InStrain also classifies the identified SNVs as synonymous, non-synonymous, or intergenic SNVs. In our benchmark testing pipeline, we ran InStrain with default parameters: minimal coverage of a position of five reads, minimal frequency of an SNP of 0.05 and an FDR (based on *a priori* empirical tests) of 1 × 10^–06^.

### Joint Variant Calling

Most variant callers pull information from a population of samples to make their calling more accurate. The same options we described above were therefore used to perform variant calling on two BAM files at the same time to test for improved performance. Each of the BAM files represented a different scenario, namely, uni-strain or multi-strain. For BCFtools, we performed a joint mpileup call followed by a BCFtools call. For freebayes, we included both BAM files in the input. In HaplotypeCaller and Mutect2, we performed classic joint calling by calling variants simultaneously across BAM. In metaSNV, we profiled both BAM files together. To the best of our knowledge, variant calling in InStrain and VarScan2 does not benefit from joint variant calling and was not done.

### Statistics Assessment

Variant calling outputs were reformatted to a homogenous format. From the VCF outputs (BCFtools, freebayes, HaplotypeCaller, Mutect2), we extracted information regarding chromosome, position, reference allele, and alternative allele. When variants of more than one nucleotide were reported, they were decoded into as many independent variants as polymorphisms found. If the Phred quality score was available, this information was included in the standardized file. Multiple alternative allele variants were encoded as independent variations. MetaSNV was similarly reformatted, and multivariate positions were decoded as independent variants. InStrain’s positions were transformed to 1-based indexing, and each nucleotide that did not match the reference was decoded as an independent variation.

In joint variant calling, for VCF files, we used the predicted genotype in each of the input files.

The set of sequence-covered variants was determined by overlapping the list of simulated true variants with the coverage profile generated by Samtools (v1.9) mpileup.

Next, the list of mutations covered was overlapped with each of the tool’s variant calls. True positives (TP) were when the variant was present in both the called profile and the covered variants. False negatives (FN) were when the covered variants were not present in the called profile. False positives (FP) were when the called variants were not present in the covered profile. True negatives (TN) were all the covered positions that remained after subtracting TPs, TNs, and FPs. Sensitivity was calculated using Equation (4) and precision using Equation (5).

$$S\,e\,n\,s\,i\,t\,i\,v\,i\,t\,y=\frac{T\,P}{\left(T\,P+F\,N\right)}$$

**Equation 4.** Sensitivity. TP, true positives; FN, false negatives.

$$P\,r\,e\,c\,i\,s\,i\,o\,n=\frac{T\,P}{\left(T\,P+F\,P\right)}$$

**Equation 5.** Precision. TP, true positives; FP, false positives.

### Receiver Operator Characteristic Curves

We generated sensitivity and precision statistics using the different Phred score thresholds provided in the probabilistic methods BCFtools, freebayes, and HaplotypeCaller. We generated 50 quality thresholds ranging from the 2% quantile to the 100% quantile of the Phred score distribution in each sample.

### Variant Calling in Real Data

Metagenomic data from the HMP (Schloissnig et al., 2013) belonging to 43 participants from samples taken at two timepoints up to a year apart (86 samples total) were downloaded from the HMP public repositories^4^. We then selected IDs based on Supplementary Table 1 from Schloissnig et al. (2013). Reads were pruned of human contamination, trimmed using KneadData, and mapped to the reference genomes of 10 representative species that we previously benchmarked on simulated data. These representative species were selected by calculating the mean sensitivity and precision statistics measured for each species in the simulation dataset across all tools. The genomes were selected to represent both good and poor SNV calling performance and the overall genetic diversity (Supplementary Figure 2). A Manhattan distance matrix was then calculated based on mean sensitivity and precision of each species. Finally, based on the calculated Manhattan distance matrix, we assigned all species into 10 clusters using the partitioning clustering pam() function in R. A representative species in each cluster was randomly selected. The representatives were merged in a unique reference and mapped against the 86 paired-end samples using Bowtie2 (Langmead and Salzberg, 2012). Picard’s MarkDuplicates and CleanSam were used to clean the mapped reads. Variant calling on BAM files was performed with HaplotypeCaller and Mutect2, as described above.

### Genetic Distance Calculation

We computed the genetic distance between pairs of HMP samples. For this, we first defined a set of variants (reference variants) by including all called variants in any taxon and sample that were present in at least two HMP samples (removing singletons). We further produced a site frequency spectrum plot by counting the number of individuals in which each variant was observed. We profiled each sample by creating a presence–absence matrix for each of the reference variants. We then computed the Manhattan distance [python scipy, spatial.distance.pdist (metric = “cityblock”)] between the different samples. We also reproduced the same analysis using only the variants found in each of the species in the reference genome.

### Clustering of Intra-Individual and Inter-Individual Samples

Genetic distances were clustered using hierarchical clustering with the nearest point algorithm. The number of samples clustering together at both baseline and follow-up were counted. In addition, we did a second clustering based on Bray–Curtis distances using the HMP abundance data generated with the previously described settings (Chen et al., 2020). The distances between the same individual at baseline and follow-up (intra-individual) and among independent samples (inter-individual) were compared using the Wilcoxon test.

### Statistical Analysis

Rv3.5.1 and Pythonv3.7.0 were used for plotting and statistical calculations. To address the effect of bacterial abundance and genome coverage on the specificity and sensitivity of the different tools, we associated the precision and sensitivity of different tools with bacterial abundance and coverage using Spearman’s correlation. The effect of genome quality on precision and sensitivity was assessed by linear modeling (ordinary least squares). We built a null model explaining either precision or sensitivity while including an interaction effect of tools and simulation design (uni-strain or multi-strain), which was found to be significant. We then built a second model including either the N50 or the number of contigs as a proxy of genome quality on top of the null model. We also built a third model that considered an interaction effect of the genome quality and tool and design. We assessed significance among the nested models using a likelihood ratio test.

To address the effect of simulation benchmark metrics and the number of variants called in a genome on real data clustering performance, we built a linear model using the percentage of samples where baseline and follow-up clustered together as the dependent variable and the tool, benchmarked sensitivity and accuracy, and number of called variants per bacteria as regressors.

We used Wilcoxon tests to estimate whether there were differences between the specificity and sensitivity statistics of joint-called samples or individually called samples. In addition, we compared sensitivity and accuracy metrics between the simulations with 1 and 4% of mutated positions using Pearson correlation.

We set a significant *p*-value threshold of 0.05.

### Data Availability

The pipelines used for simulation and variant calling in simulated data and for variant calling in real data were written in Snakemake (v5.9.1) (Köster and Rahmann, 2012) and can be found, together with the plotting and statistical analyses scripts, in our Github repository^5^.

## Results

### Experimental Design and Simulations

We first simulated metagenomic datasets. We did so by including the 45 most common bacterial species, which accounted for an average 74% of bacterial abundance in a recent large multi-ethnic study (Gupta et al., 2020) and adding one genome with the remaining abundance to reach 100%. The reference genomes for the 46 species were randomly selected from the species genomes available in the NCBI. We then introduced known SNV variants in 1% of the genomic positions. On average, the number of contigs found in each reference genome was 112.17 and ranged between 1 and 1,541 (Supplementary Figure 1), and the average N50 was 1,297,250.67 base pairs (5,884–6,271,157) (Supplementary Figure 1). The N50 distribution of all reference genomes followed a bimodal distribution, showing the existence of both high- and low-quality reference genomes.

To address the presence of homology among species, which may bias read mapping, we measured the k-mer-based distance of the reference genomes using Mash (Ondov et al., 2016). We found a mean Mash distance of 0.35 (0.04–1, *SD* = 0.08) (dendrogram shown in Supplementary Figure 2). In addition, we counted the number of reads that mapped equally well in more than one position to a concatenated multi-reference genome and found 8% of reads to be multi-mappers. However, if we consider discordant pairs as incorrectly assigned reads due to homology or horizontal gene transfer events, this percentage grows to nearly 36% (if multi-mappers are also considered), which will influence the false positives identified by SNV-calling tools.

**FIGURE 1:**
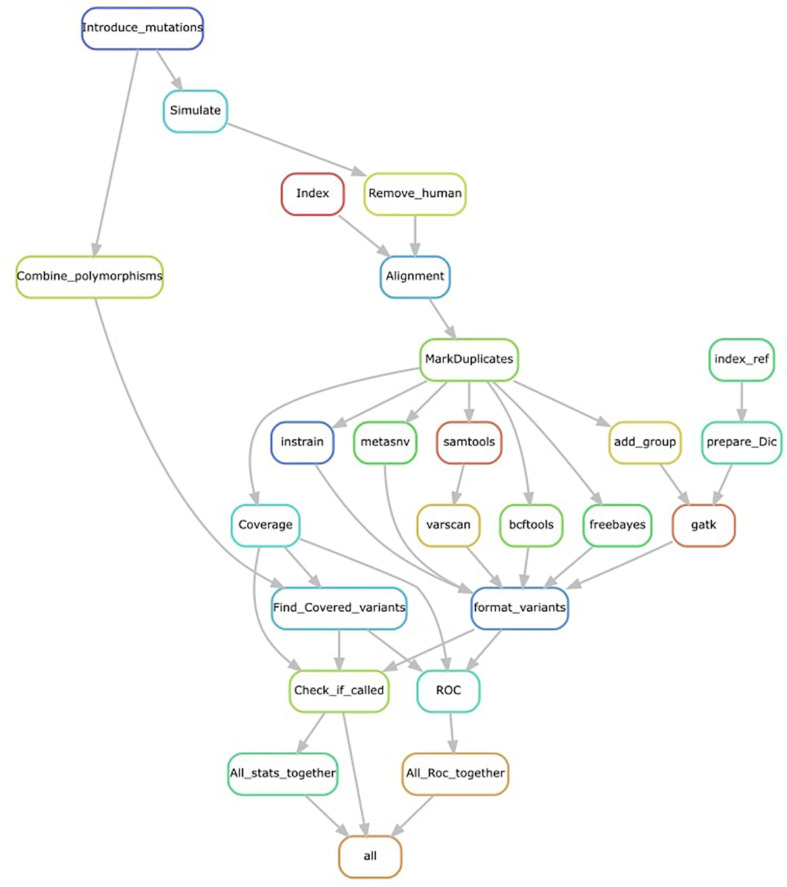
Representation of the pipeline used. Mutations are introduced into each reference genome before simulation. Simulated reads are trimmed, and human contamination removed. Reference genomes are indexed and mapped using simulated cleaned reads. Alignments are further cleaned before variant calling. All variant outputs are converted to the same format. Introduced variants covered by simulated reads are used as a set of true positives. Variants are checked in the formatted variant calls, and receiver operator characteristic (ROC) curves are calculated by repeating this process at different quality thresholds. All statistics are combined in a single file.

We processed the simulated reads (using KneadData for trimming and Bowtie2 for mapping) and ran the seven different variant-calling tools (Figure 1). Of these, four are probabilistic methods—BCFtools, Mutect2, HaplotypeCaller, and freebayes—meaning that they use the coverage, base call quality, and error rate expectations to infer genotype likelihoods and call non-reference nucleotides. We used options for haploid variant calling and, as a metagenome, can be considered as a pooled sample of an unknown number of multiple organisms; if an option for running an unknown number of pooled samples existed, we used it (e.g., in freebayes). The other three tools—VarScan2, metaSNV, and InStrain—are non-probabilistic methods and are mainly based on applying specific filters as the minimal coverage to consider a variant, which we set to the default value for each tool (Table 1).

We ran two different simulations, one assuming that one unique strain was present per bacterial taxa (uni-strain scenario) and another assuming two different strains per bacterial taxa (multi-strain scenario).

### Probabilistic Methods Show Better Sensitivity and Precision

**FIGURE 2:**
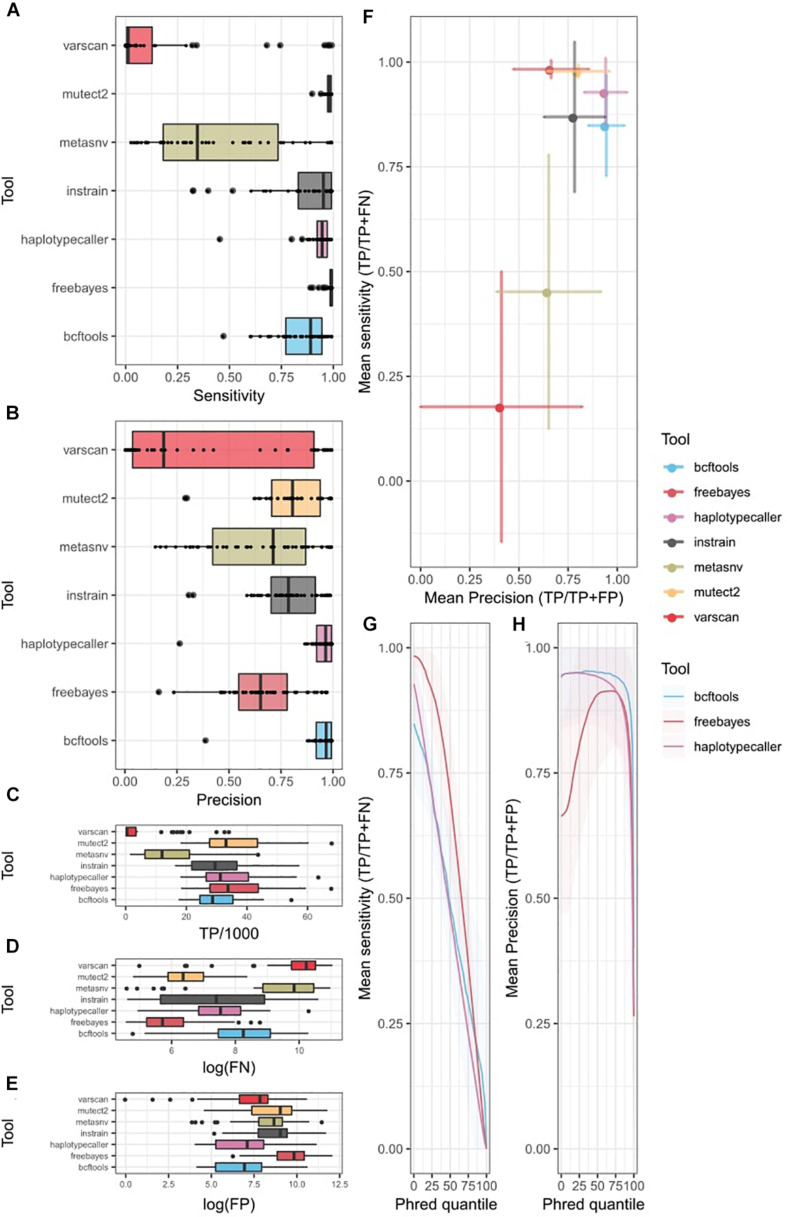
Single strain variant-calling statistics of seven different tools. Colors indicate different tools. **(A)** Sensitivity (TP/TP + FN) of each tool. Tukey box plot presents the distribution of precision. Dots show precision per individual bacteria. **(B)** Precision (TP/TP + FP) of each tool. Tukey’s box plot presents the distribution of precision. Dots show precision per individual bacteria. Distribution of **(C)** TP, **(D)** FN, and **(E)** FP per tool as Tukey box plots. Individual dots indicate bacteria over 1.5 times the interquartile distance. **(F)** Precision vs. sensitivity plot. Dots indicate mean values among all bacteria. Error bars represent the standard deviation from the mean. **(G,H)** ROC curve of probabilistic methods. *X*-axis represents the quantile Phred filter. **(G)** Mean sensitivity changes with changes in Phred score. Line shows the mean value among bacteria. Shading represents the standard deviation from the mean. **(H)** Mean precision changes with Phred score changes. Line represents mean value among bacteria. Shading represents the standard deviation from the mean. TP, true positives; FP, false positives; FN, false negatives.

We computed sensitivity and precision statistics from the variant-calling results (Supplementary Table 3). In the uni-strain scenario, all four probabilistic methods showed high sensitivity for most of the organisms (Figure 2A). Mutect2 and freebayes had the highest sensitivity, recalling nearly 100% of covered variants, followed by HaplotypeCaller and InStrain (the only non-probabilistic method with high sensitivity), which had low sensitivity for some taxa. BCFtools ranked 5th, and there was a large difference in performance between metaSNV, which showed the largest variability among taxa, and VarScan2, which had poor sensitivity in most samples. Both metaSNV and VarScan2 missed many covered variants (Figures 2C,D). Precision was also higher in the probabilistic tools (Figure 2B), where both BCFtools and HaplotypeCaller achieved a similar performance, with a low number of FP (Figure 2E) and little variation in precision among taxa compared with other tools. They were followed by Mutect2 and InStrain. MetaSNV showed a better average precision than freebayes, but also had a higher standard deviation. Once again, despite its low number of FN, VarScan2 was penalized by its low number of TP and showed the highest variation and average low precision.

Overall, probabilistic methods showed the best compromise between sensitivity and precision (Figure 2F). They showed a lower precision and sensitivity variability among taxa compared with non-probabilistic methods, which were penalized by unequal coverage in low-abundance species. This was especially true when comparing sensitivity. However, the most sensitive tools, freebayes and Mutect2, showed high variability in their precision. HaplotypeCaller, with the highest precision, seems a better option than BCFtools, which had a lower sensitivity despite having a similar precision.

In addition, we tested tool performance by including more divergent strains from the reference (4%) in a uni-strain scenario. These results replicated our observations from the 1% divergence scenario (Supplementary Figure 3) and showed an overall high correlation coefficient both in sensitivity and precision (Supplementary Table 4).

An additional advantage of probabilistic methods is the availability of a quality metric that can be easily tuned to recalculate sensitivity and precision values (Table 1). We generated an ROC curve for different values of this quality in freebayes, HaplotypeCaller, and BCFtools (Figures 2G,H) and observed an almost linear decrease in sensitivity with higher-quality thresholds in all three tools. freebayes remained the most sensitive method at almost any quality threshold. BCFtools achieved a better sensitivity than the other two tools at higher-quality thresholds, which seems to indicate that the highest quality values of freebayes and HaplotypeCaller have, on average, more FP. The precision curve showed large variability among bacteria. Both BCFtools and freebayes, which showed the highest precision without any tuning, did not improve their precision with higher thresholds, on average. However, their precision did decrease at the highest thresholds, probably showing the existence of FP when using a high-quality threshold. On the other hand, freebayes’ precision was improved substantially by increasing the quality threshold, but needs up to a 75% quantile, on average, to catch up to the other tools, which results in an acute decrease in sensitivity. However, given the large number of low-quality calls in freebayes (Supplementary Figure 4), a minimal quality filter may be required to substantially improve the performance of this tool.

We further explored the sensitivity and precision of tool performances in the multi-strain scenario (Supplementary Table 5). Since both strains only differed in the genomic locations where the 1% of variants were generated, no structural or copy number variations were included. The major difference compared with the uni-strain scenario was that there were twice as many variants. Therefore, there might be multiple variants in the same locus, and the number of reads covering a single variant from a specific strain were now reduced by half (both strains are assumed to have equal abundance, so each has half the abundance simulated in the uni-strain scenario).

Sensitivity showed an acute decrease (Supplementary Figure 5). While some tools achieved a mean sensitivity of ∼90% in the uni-strain case, in the multi-strain scenario, this value was only achieved for some species. Such species did not have a significantly higher abundance than the ones with lower sensitivity. Mutect2 and freebayes remained the two tools with the highest recall, with median sensitivity around 50%, which might be explained by the fact that the most-covered positions might not include the variant from one of the strains but rather the reference allele from the other strain. This could increase the FNs, despite the mutation not being covered. The precision results showed the same pattern and range as in the single-strain scenario since FNs are not used, showing high estimates mainly in HaplotypeCaller and BCFtools. ROC curves in BCFtools, freebayes, and HaplotypeCaller also showed similar results to the uni-strain scenario.

### Joint Variant Calling Benefits Complex Scenarios With Multiple Strains

**FIGURE 3:**
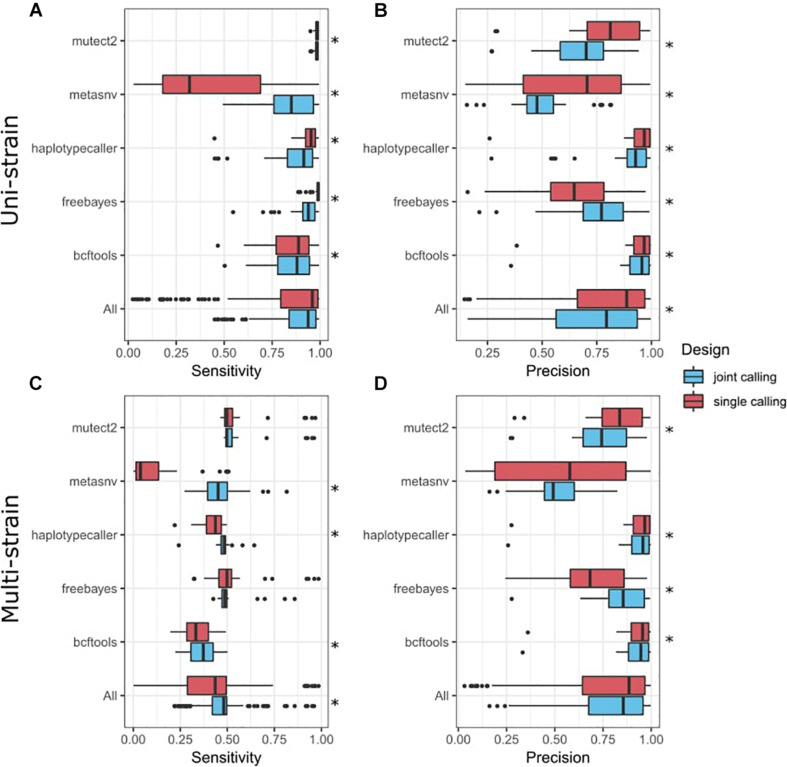
Comparison of variant-calling performance between joint calls and single calls. *Y*-axis presents tools, including a combination of all tools (All). Tukey box plots are shown and colored according to the variant-calling mode. Single points represent samples >1.5 times the interquartile distance. Asterisks indicate statistically significant differences (*p* < 0.05) in a paired Wilcoxon test comparing both groups. **(A)** Sensitivity metrics in the uni-strain scenario. **(B)** Precision metrics in the uni-strain scenario. **(C)** Sensitivity metrics in the multi-strain scenario. **(D)** Precision metrics in the multi-strain scenario.

Most of the tools analyzed also enable joint variant calling of multiple samples, meaning that different samples (in our case simulations) are pooled together during the variant-calling process, in contrast to the independent variant calling in each individual sample that we had performed previously. This could improve overall performance, although it would make it more difficult to detect singletons. We combined our two simulated datasets in order to perform joint variant calling in the tools where we expected this would be beneficial: BCFtools, freebayes, Mutect2, HaplotypeCaller, and metaSNV (Figure 3). Our results show that joint variant calling increased the sensitivity in the multi-strain scenario (*p* = 3.68 × 10^–18^) (Figure 3C). Precision was also significantly improved in freebayes, while Mutect2, BCFtools, and HaplotypeCaller had a significant decrease in precision (Figure 3D). In the uni-strain scenario, joint calling only improved metaSNV’s sensitivity (Figure 3A) and freebayes’ precision (Figure 3B) and led to an overall decrease in both precision and sensitivity.

### Factors Affecting Variant-Calling Performance: Species Abundance and Coverage Have a Tool-Specific Effect, Whereas the Effect of Genome Quality Is Constant

**FIGURE 4:**
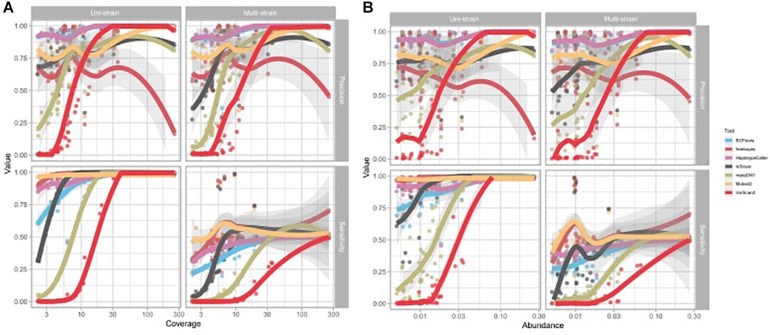
Effect of average depth of genome coverage and species abundance on variant calling performance. Each dot represents a sample. **(A)** Effect of reference genome coverage on precision and sensitivity of each tool. **(B)** Effect of species abundance on precision and sensitivity of each tool. Trend lines were fitted with local polynomial regression (LOESS). Shading represents the 95% confidence interval of the trend line.

The precision and sensitivity of SNV calling is affected by both species’ abundance and coverage, with the non-probabilistic tools VarScan2 and metaSNV especially affected (Figure 4). For species with low or medium abundance, the precision and sensitivity of metaSNV and VarScan2 were lower than for other tools, and the performance of these two tools improved linearly as species coverage increased. For species with a low abundance, the sensitivities of InStrain and BCFtools were significantly affected by the coverage. The performances of HaplotypeCaller and Mutect2 were not significantly affected by species abundance and coverage: their precision and sensitivity were high and stable even in low abundance species and in both uni- and multi-strain settings. The performance of freebayes was unstable and not linearly associated with species abundance and coverage (Supplementary Table 6).

In addition, we tested if the reference genome chosen has a significant effect on tool performance using two different proxies of genome quality. First, we tested the tools with the classic N50 metric and found no significant effect on either sensitivity or precision metrics. The number of contigs per reference did have an overall significant negative effect on sensitivity (*p* = 8.42 × 10^–6^) and precision (*p* = 3.76 × 10^–9^), but there was no significant interaction effect with specific tools.

### Genetic Distances Are More Individual Specific Than Bacterial Abundance

**FIGURE 5:**
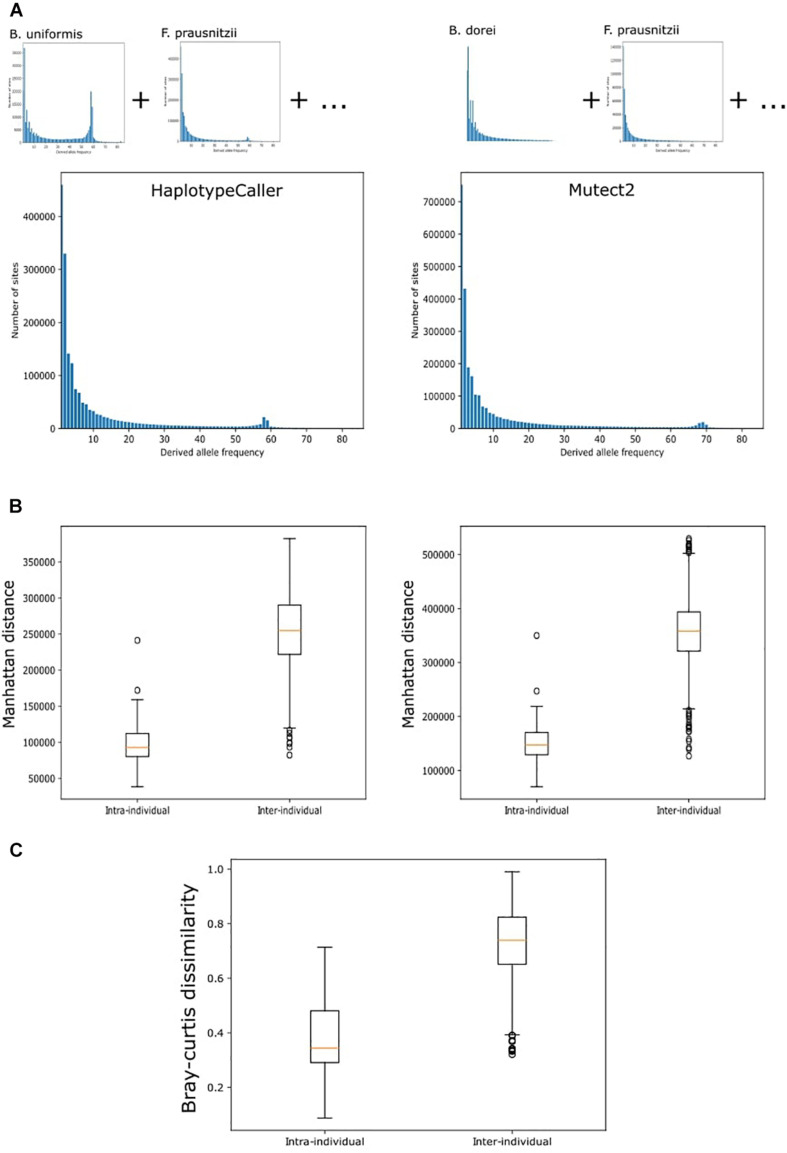
Variant calling on real data. **(A)** Site frequency spectrum of variants in the whole metagenome called by HaplotypeCaller and MetaSNV. Total site frequency spectrum is the sum of the variants in the 10 chosen bacterial taxa. **(B)** Genetic distance between samples. Manhattan distances were calculated from the SNV profile of each sample. Tukey boxplot shows the distribution of distances between samples belonging to the same individual at two timepoints (intra-individual) and between samples from different people (inter-individual). These distances were used to cluster the data. **(C)** Tukey boxplot of the distribution of Bray-Curtis dissimilarities computed from the estimation of taxonomic abundance between samples from the same individual and from different people.

Finally, we decided to apply the best performing tools to real HMP data from 43 individuals taken at two timepoints up to 1 year apart (Schloissnig et al., 2013). For this, we chose Mutect2, which showed the best sensitivity under all conditions tested, and HaplotypeCaller, which showed the best precision and a better sensitivity than BCFtools. The overall genetic distance between samples was estimated from the combined SNV profile from 10 selected bacteria. The number of variants identified with both methods included a large number of singletons (Figure 5A). We therefore considered only variants observed in at least two samples. The genetic distance between individuals in both Mutect2 and HaplotypeCaller, as measured by Manhattan distance, showed that samples from the same individual clustered together in 93% of cases (40 of 43 samples) (Table 2). Using individual bacteria instead of the combined genetic distance, the clustering values ranged from 4.6% (*Eubacterium hallii*, both tools) to 97% (*Bacteroides uniformis*, using Mutect2) (Table 2). Bacteria with higher resolution potential showed no correlation with simulation scores but had a positive association with the number of called variants (lineal model, *F*-test, *p* = 5 × 10^–4^). Using the complete variant dataset, we found a highly significant difference in the distribution of distances between intra-individual and inter-individual samples (Wilcoxon test, HaplotypeCaller: *p* = 1.31 × 10^–28^, Mutect2: *p* = 1.51 × 10^–28^) (Figure 5B). These results did not improve when we only considered variants present in both methods at the same time. In addition, we performed the same analysis based on taxonomic abundance, where we could cluster together 63.7% of the samples (27 of 43) (Figure 5C), highlighting the stability of genetic variation in comparison with taxonomic abundance.

**TABLE 2:** Single nucleotide variation (SNV) profiles in the Human Microbiome Project (HMP) data.

Taxa_profiled	Tool	Number of variants	% Clustered	Sensitivity	Precision
*Akkermansia_muciniphila*	M	132,878	20.9	0.955	0.992
*Akkermansia_muciniphila*	H	115,968	18.6	0.952	0.996
*Alistipes_shahii*	M	180,781	74.4	0.984	0.852
*Alistipes_shahii*	H	98,442	69.8	0.951	0.976
*Bacteroides_dorei*	M	264,480	93	0.968	0.290
*Bacteroides_dorei*	H	225,677	93	0.453	0.263
*Bacteroides_uniformis*	M	272,535	97.7	0.994	0.705
*Bacteroides_uniformis*	H	218,598	93	0.957	0.986
*Dorea_formicigenerans*	M	120,086	16.3	0.974	0.682
*Dorea_formicigenerans*	H	77,326	7	0.879	0.881
*Eubacterium_hallii*	M	75,369	4.7	0.989	0.802
*Eubacterium_hallii*	H	48,786	4.7	0.963	0.964
*Eubacterium_rectale*	M	234,000	79.1	0.953	0.830
*Eubacterium_rectale*	H	189,376	65.1	0.949	0.993
*Faecalibacterium_prausnitzii*	M	324,595	46.5	0.972	0.931
*Faecalibacterium_prausnitzii*	H	217,101	37.2	0.970	0.995
*Ruminococcus_gnavus*	M	86,590	23.3	0.983	0.738
*Ruminococcus_gnavus*	H	52,391	16.3	0.922	0.910
*Ruminococcus_sp_5_1_39BFAA*	M	182,558	34.9	0.964	0.799
*Ruminococcus_sp_5_1_39BFAA*	H	107,252	14	0.938	0.968
Total SNV profile	M	1,873,872	93	NA	NA
Total SNV profile	H	1,350,917	93	NA	NA

**Taxa profiled indicates the name of each of the chosen bacteria profiled. Total SNV profile refers to the total profile, including all variants. Tool indicates the variant caller used: M, Mutect2; H, HaplotypeCaller. Number of variants indicates the total number of variants uncovered with presence in at least two samples. % Clustered indicates the percentage of samples that clustered together at both follow-up and baseline. Sensitivity and precision are the statistics estimated from the uni-ref simulation.**

## Discussion

Microbiome genomic analyses are currently complicated by several factors, including low taxon-specific read depth, unequal taxonomic abundance, the existence of orthologs and paralogs, and horizontal transfer of genetic material. On top of these issues, single nucleotide variant calling suffers from the lack of high-quality reference genomes and the pooling of a population consisting of an unknown number of genomes. This benchmark study therefore assessed the performance of current variant callers in this complex scenario.

We used a homogenous pipeline that does not consider the complexity layer of read mapping since we used the default bowtie2 options. We used 45 microbial species that are highly abundant and prevalent in the human gut (Gupta et al., 2020) to create two simulation datasets that mimic HiSeq MGS experiments. Reference genomes for each of the species were randomly selected from GenBank and contained both high- and low-quality assemblies. Although the number of contigs present in the assembly, which might indicate genome fragmentation and poorer assemblies, did correlate with an overall decrease in sensitivity and precision, this effect was not tool specific and should not bias our comparison. This does, however, indicate that genome quality is an important factor to consider in the variant calling processing. In this line, it is important to highlight that previous benchmarks of bacterial variant calling have shown that reference selection is a crucial step (Bush et al., 2020): greater genetic distance between the sequenced strain and the reference leads to poorer variant-calling performance. One possible approach to improve the accuracy of genetic analyses of the microbiome is to use metagenomic assembled contigs from the studied metagenome as the reference. For example, Lou et al. (2021) recently used this approach coupled with InStrain variant calling, and it can be applied with any of the variant-calling methodologies we describe here. On the other hand, taxonomic abundance, which is related to the mean coverage of the genome, does influence variant-calling performance. This is especially true in the non-probabilistic methods that rely on hard cutoffs for the number of reads supporting a variant. In practice, this threshold might be optimized according to the bacterial abundance and number of reads, but we used default threshold parameters for the purposes of this work.

We chose to benchmark four commonly used probabilistic variant callers: BCFtools, Mutect2, HaplotypeCaller, and freebayes. We also included VarScan2 because it performs well for pooled samples and in circumstances where probabilistic methods do not work. We also chose to test InStrain and metaSNV as representatives of variant callers developed specifically for metagenomic datasets. Of these tools, freebayes, Mutect2, metaSNV, and InStrain are also able to identify variants from a population of samples, as is the case if several strains coexist, or homologous regions from different taxa align. Variant calling was performed independently in each simulation set and bacteria, as was mapping. However, this might not be ideal for some tools. InStrain, for instance, recommends mapping to a database containing all reference genomes so that multi-mapping reads will be penalized with lower mapping quality. VarScan2 also relies on mapping quality trimming, which penalizes multi-mapping reads.

Our simulations consisted of two scenarios. In the first, only one strain per species was simulated. This might correspond to the real gut metagenomic data, since one major strain dominates the environment in many cases (Truong et al., 2017). In the second scenario, we assumed the existence of two strains with equal abundance per species. Both strains were simulated as only containing SNVs and with no other structural variations, which is an important simplification to consider when looking at our results. Our performance estimates only considered positions covered by reads, and thus, if most variants were missed due to a lack of coverage, we could not consider them. This is a double-edged sword because, in the multi-strain scenario, positions might be covered by one strain that does not contain the variant, and we will thus overestimate the FN fraction compared with the uni-strain scenario. Consistent with this expectation, our sensitivity results here are about half of those achieved in the uni-strain scenario. Nonetheless, the tool comparisons in both scenarios are similar. Most tools achieved high precision, particularly BCFtools and HaplotypeCaller. Both these methods are probabilistic and do not consider population variants, which means that the calls are more restrictive (no multiple alleles are expected in a haploid genome) but have more information to successfully call true variants. On the other hand, freebayes and Mutect2 achieved higher sensitivity, consistent with their ability to detect multiple variants per locus. These results highlight that, while non-probabilistic methods have been developed to deal with the issues associated with MGS variant calling, probabilistic methods can still perform better or similarly, at least when analyzing very abundant bacteria. However, we also show that the performance of non-probabilistic methods declined drastically for lower abundance bacteria. This might highlight the necessity of fine tuning the default threshold values according to the genome size and the number of reads produced. This is especially true for VarScan2, where default values are not tuned for metagenomic calling and resulted in very restrictive cutoffs that reduced the number of calls.

In addition, we also tested the differences in performance of HaplotypeCaller, freebayes, and BCFtools, which all give Phred-score quality values for their variant callers. Our results highlighted that the highest-scoring variants tended not to be TP and might indicate homologous regions with other bacteria. At the same time, only freebayes benefited substantially from quality filtering, which improved its precision as most of the variants found were of very low quality.

Joint variant calling of the uni-strain and multi-strain scenarios improved sensitivity in relation to non-joint variant calling. However, joint variant calling negatively affected the uni-strain results. As it is difficult to assess which situation is most likely to occur in real data and, given the good performance of non-joint variant calling in our simulation, we advocate performing SNV calling per sample instead of joint calling.

Finally, we investigated real gut metagenomic data from the HMP where we did not have certainty about which variants are true or false. However, given the longitudinal sampling of these HMP samples, we could use our variant set to compare samples at baseline and follow-up, assuming that most genetic variants would be stable within 1 year. Here we chose only 10 species for variant calling so that representatives of the different performances in the simulated data were used. Variants were profiled with two tools, HaplotypeCaller, which had the best precision in our benchmark, and Mutect2, which had the best sensitivity. Both tools showed good performance even for low-abundance species. Our results show that both methods we used to call HMP variants produced variant profiles that were closer between samples taken from the same individual at different times than among different individuals. In fact, we were able to demonstrate that this individual specificity is even higher than abundance-based estimations.

Variant-calling errors are expected to arise with lower read depth [due to the relative abundance of a given taxon or systematic bias during sequencing protocols (Browne et al., 2020)], with lower sequencing quality in certain regions [due to inherent sequencing biases that are platform dependent (Ross et al., 2013)], and with wrongly mapped reads (possibly in low-complexity or homologous regions), which have a fundamental role in variant-calling performance. Of these potential sources of bias, we assessed the effect of relative abundance. However, all our simulations follow an Illumina error model, which does not account for genomic features prone to generate sequencing errors, except for errors related to read position. With respect to incorrectly assigned reads, we give an estimate of 36%, but further efforts are needed to assess to what extent these incorrectly assigned reads impact the variant calling results. Furthermore, our simulation assumed that all introduced variants were neutral and occurred by chance and did not take evolutionary forces into consideration. To verify the SNV calling from short-read MGS data, variants might be confirmed with whole-genome sequencing from single-strain isolates.

Overall, this benchmark highlights the efficacy of using probabilistic variant callers on metagenomic data. We recommend using GATK’s HaplotypeCaller or Mutect2 depending on concerns about FP (use HaplotypeCaller) or FN (use Mutect2). Both tools seem to perform equally well in real data, where we found a similar power to cluster follow-up samples.

## Data Availability Statement

Publicly available datasets were analyzed in this study. This data can be found here: https://hmpdacc.org/hmp/.

## Author Contributions

SA-S, LC, DW, and JF contributed to the project conception. SA-S, LC, DW, and HA ran the tools to complete the project. SA-S, LC, and DW conducted the formal analysis and visualization. SA-S, LC, DW, HA, and JF wrote the study, while AZ edited the manuscript. AZ and JF supervised the completion of the study. All authors contributed to the article and approved the submitted version.

## Conflict of Interest

The authors declare that the research was conducted in the absence of any commercial or financial relationships that could be construed as a potential conflict of interest.

## Footnotes

1 https://samtools.github.io/BCFtools/call-m.pdf

2 https://gatk.broadinstitute.org/hc/en-us/articles/360036712151-HaplotypeCaller

3 https://gatk.broadinstitute.org/hc/en-us/articles/360037593851-Mutect2

4 https://hmpdacc.org/hmp/

5 https://github.com/GRONINGEN-MICROBIOME-CENTRE/Groningen-Microbiome/tree/master/Projects/Metagenomics_SNVcalling_Benchmark

## Supplementary Material

Supplementary Figure 1 Distribution of genome quality. **(A)** Distribution of number of contigs. **(B)** Distribution of N50 score. **(C)** Distribution of auN. **(D)** Distribution of genome length. Vertical line represents mean value.

Supplementary Figure 2 Dendrogram of Mash-based distance between the 46 reference genomes used in the benchmark. Highlighted taxa are the ones picked for the variant calling in the HMP data.

Supplementary Figure 3 Uni-strain variant calling statistics of the seven tools on a 4% divergence set-up. Colors indicated the tools. **(A)** Sensitivity (TP/TP + FN) of each tool. Tukey’s box plot shows the distribution of precision. Dots show precision per individual bacteria. **(B)** Precision (TP/TP + FP) of each tool. Tukey’s box plot shows the distribution of precision. Dots show precision per individual bacteria. Distribution of **(C)** TP, **(D)** FN, and **(E)** FP per tool, shown as Tukey’s box plots. Individual dots present bacteria >1.5 times the interquartile distance. **(F)** Precision vs. sensitivity plot. Dots present mean values among all bacteria. Error bars represent the standard deviation from the mean.

Supplementary Figure 4 Variant Phred quality distribution in each of the 46 analyzed species in BCFtools, HaplotypeCaller and Mutect2.

Supplementary Figure 5 Two-strain variant calling statistics of the seven tools. Colors indicated the tools. **(A)** Sensitivity (TP/TP + FN) of each tool. Tukey’s box plot shows the distribution of precision. Dots show precision per individual bacteria. **(B)** Precision (TP/TP + FP) of each tool. Tukey’s box plot shows the distribution of precision. Dots show precision per individual bacteria. **(C)** Precision vs. sensitivity plot. Dots present mean values among all bacteria. Error bars represent the standard deviation from the mean. Distribution of **(D)** TP, **(E)** FN, and **(F)**. FP per tool, shown as Tukey’s box plots. Individual dots present bacteria >1.5 times the interquartile distance.

Supplementary Table 1 Table of species selected from Gupta et al., 2020.

Supplementary Table 2 Table with species, abundance, reference and genome quality metrics.

Supplementary Table 3 Variant calling statistics in the uni-strain scenario.

Supplementary Table 4 Variant calling statistics in the uni-strain scenario including 4% variation from each taxon to the reference. Includes Pearson correlation coefficients between accuracy and sensitivity from the uni-strain scenario between a simulation of 1 and 4% of mutations.

Supplementary Table 4 Variant calling statistics in the multi-strain scenario.

Supplementary Table 5 Summary statistics of the effect of genome abundance and coverage on variant calling.
