# Supplementary figures and images for: A Benchmark of Genetic Variant Calling Pipelines Using Metagenomic Short-Read Sequencing
Source: Front Genet. 2021 May 10;12:648229. doi: 10.3389/fgene.2021.648229 (PMC8141913; doi:10.3389/fgene.2021.648229)

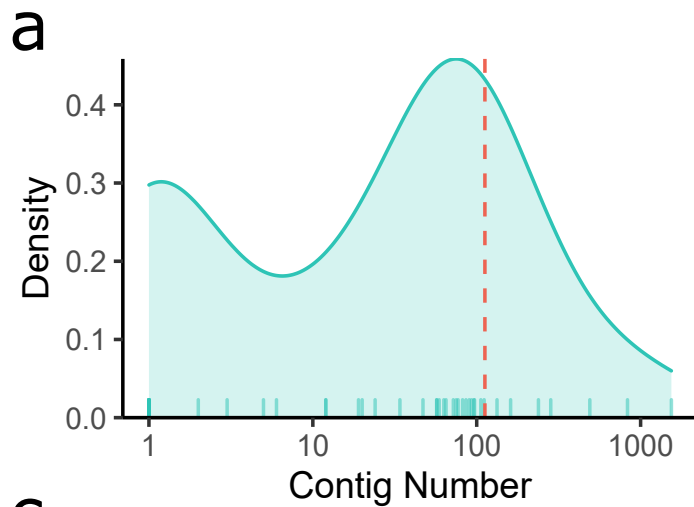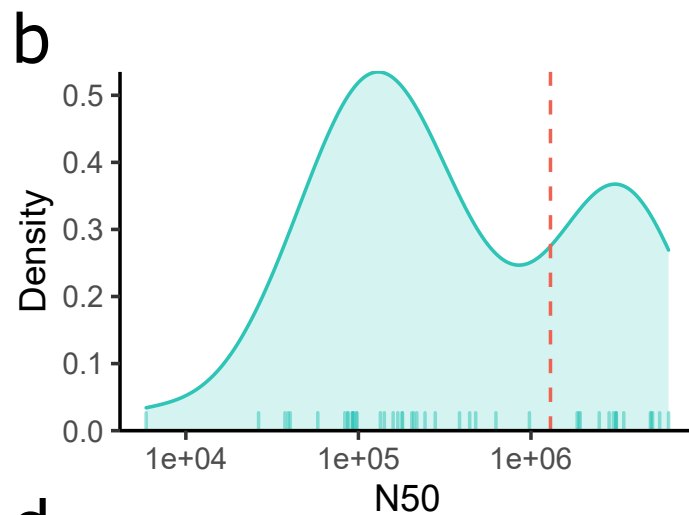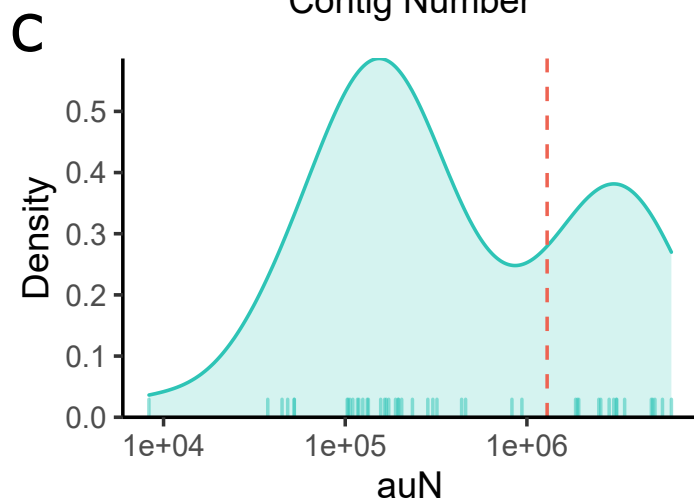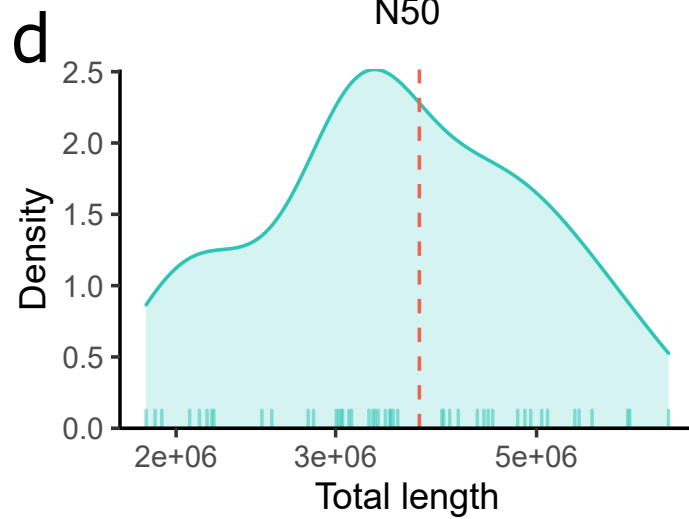

Supplement: Supplementary Figure 1 — Distribution of genome quality. (A) Distribution of number of contigs. (B) Distribution of N50 score. (C) Distribution of auN. (D) Distribution of genome length. Vertical line represents mean value. [file Data_Sheet_1.PDF]

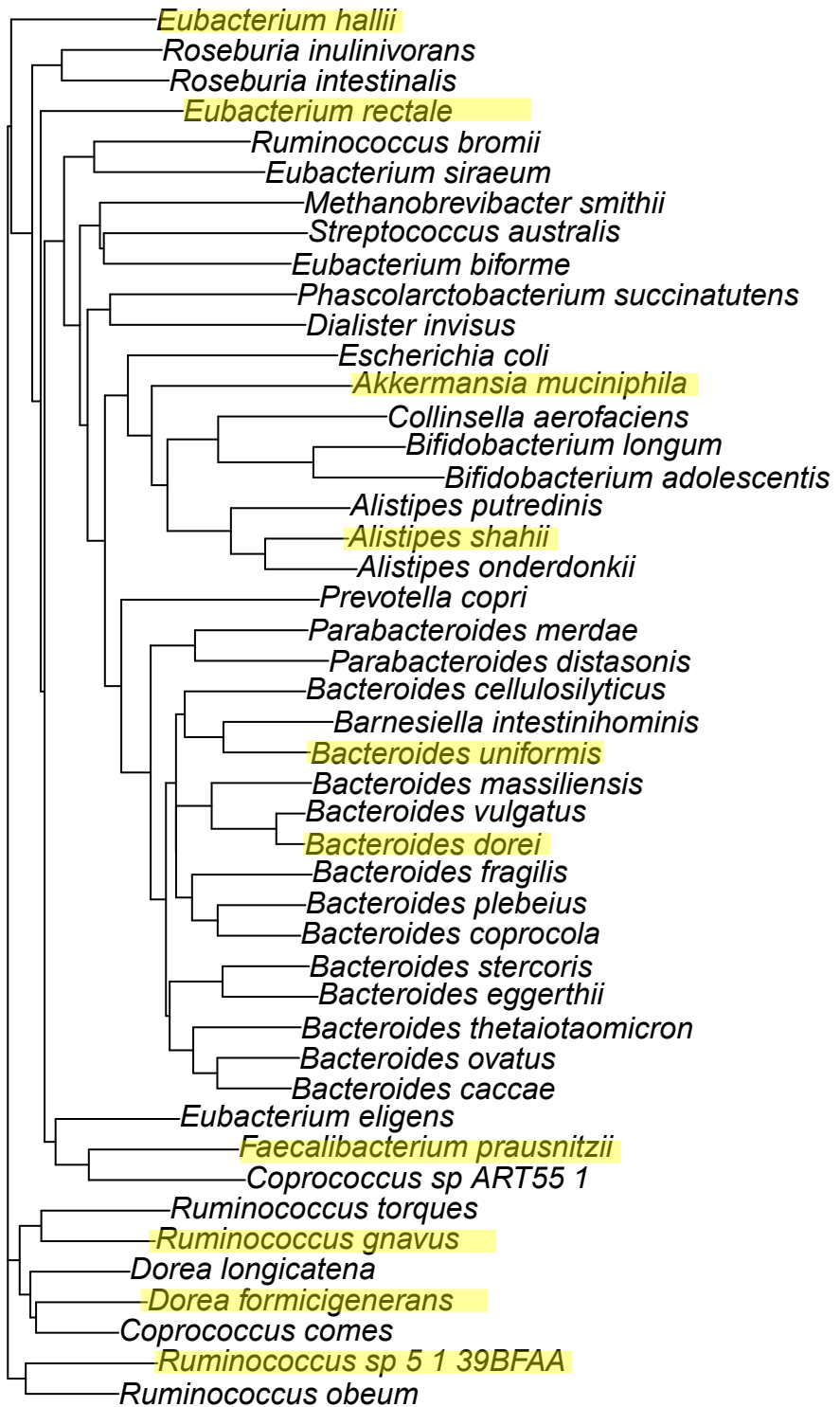

Supplement: Supplementary Figure 2 — Dendrogram of Mash-based distance between the 46 reference genomes used in the benchmark. Highlighted taxa are the ones picked for the variant calling in the HMP data. [file Data_Sheet_2.PDF]

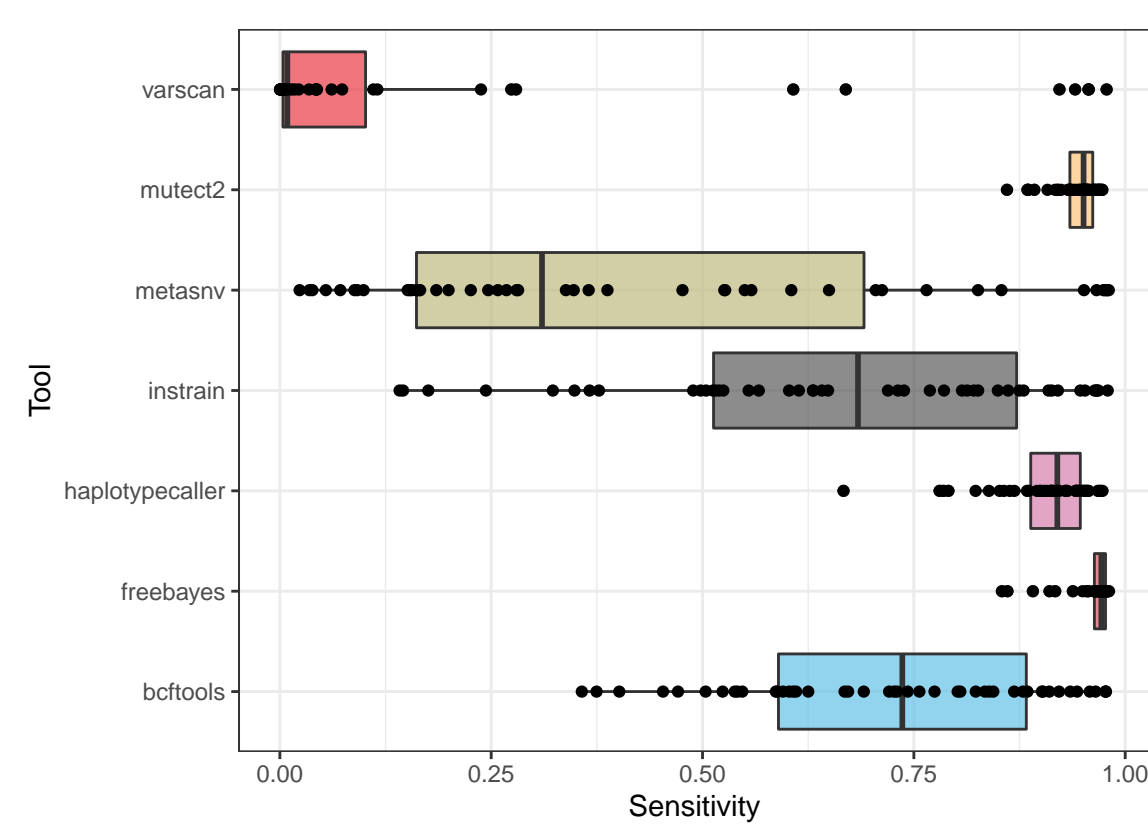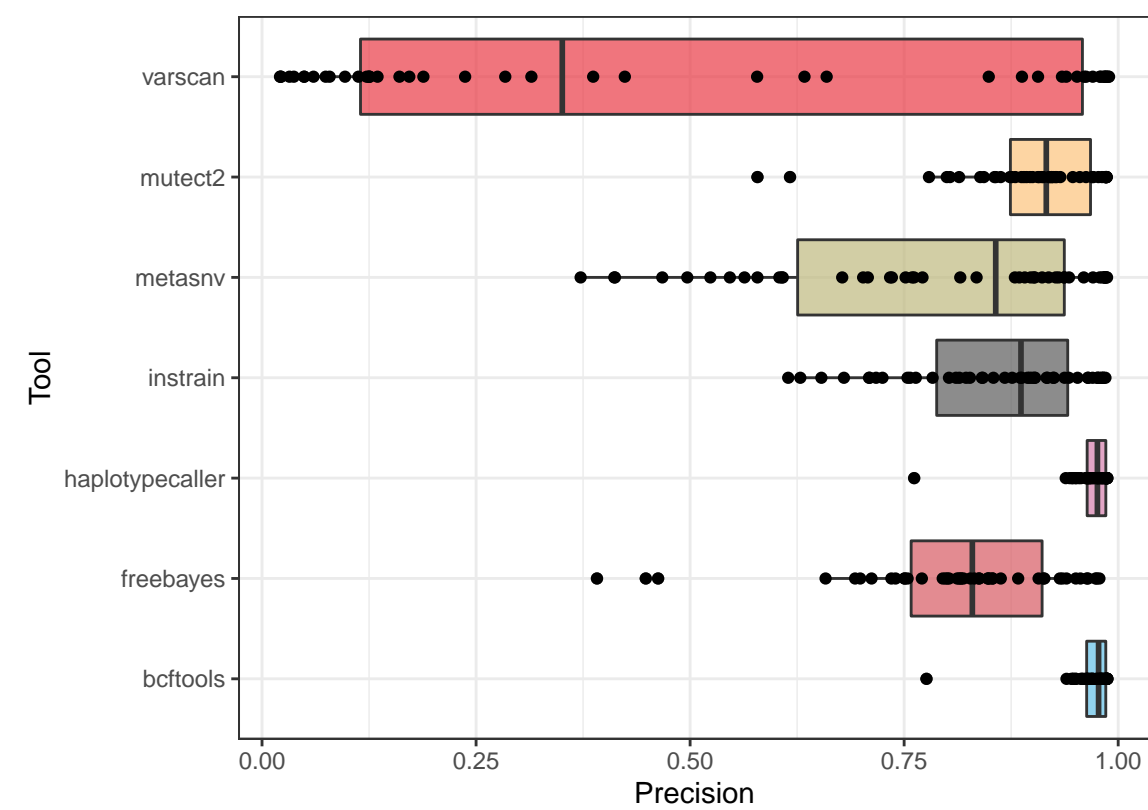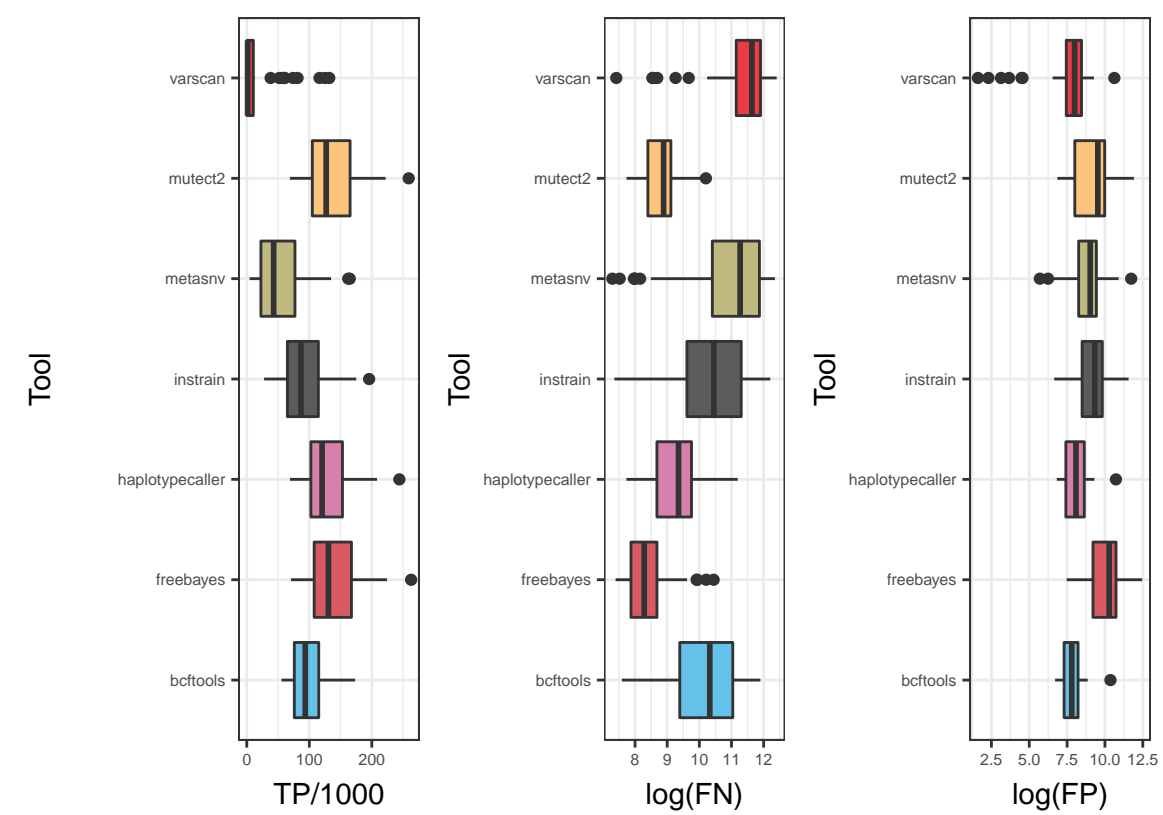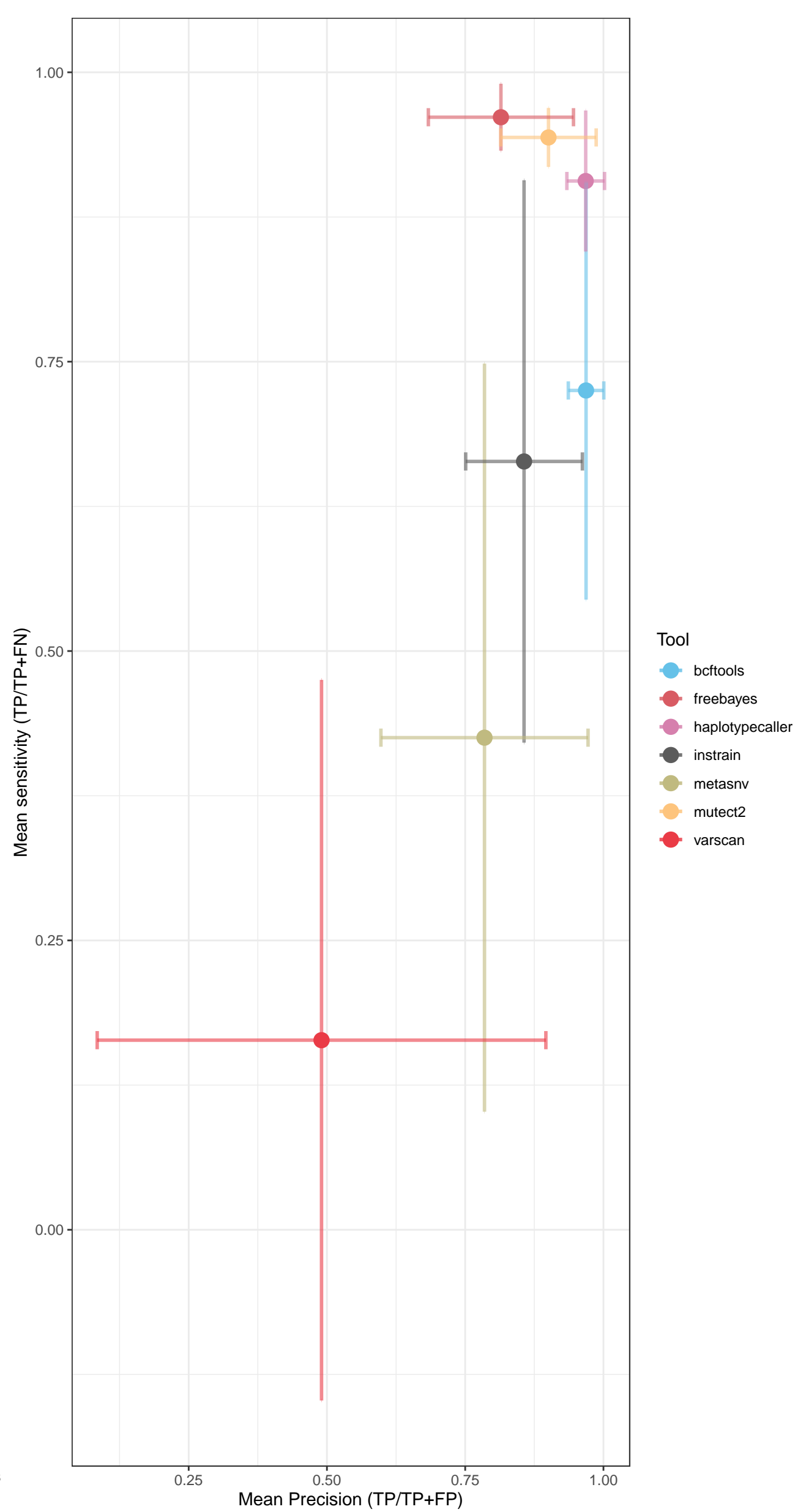

Supplement: Supplementary Figure 3 — Uni-strain variant calling statistics of the seven tools on a 4% divergence set-up. Colors indicated the tools. (A) Sensitivity (TP/TP + FN) of each tool. Tukey’s box plot shows the distribution of precision. Dots show precision per individual bacteria. (B) Precision (TP/TP + FP) of each tool. Tukey’s box plot shows the distribution of precision. Dots show precision per individual bacteria. Distribution of (C) TP, (D) FN, and (E) FP per tool, shown as Tukey’s box plots. Individual dots present bacteria >1.5 times the interquartile distance. (F) Precision vs. sensitivity plot. Dots present mean values among all bacteria. Error bars represent the standard deviation from the mean. [file Data_Sheet_3.PDF]

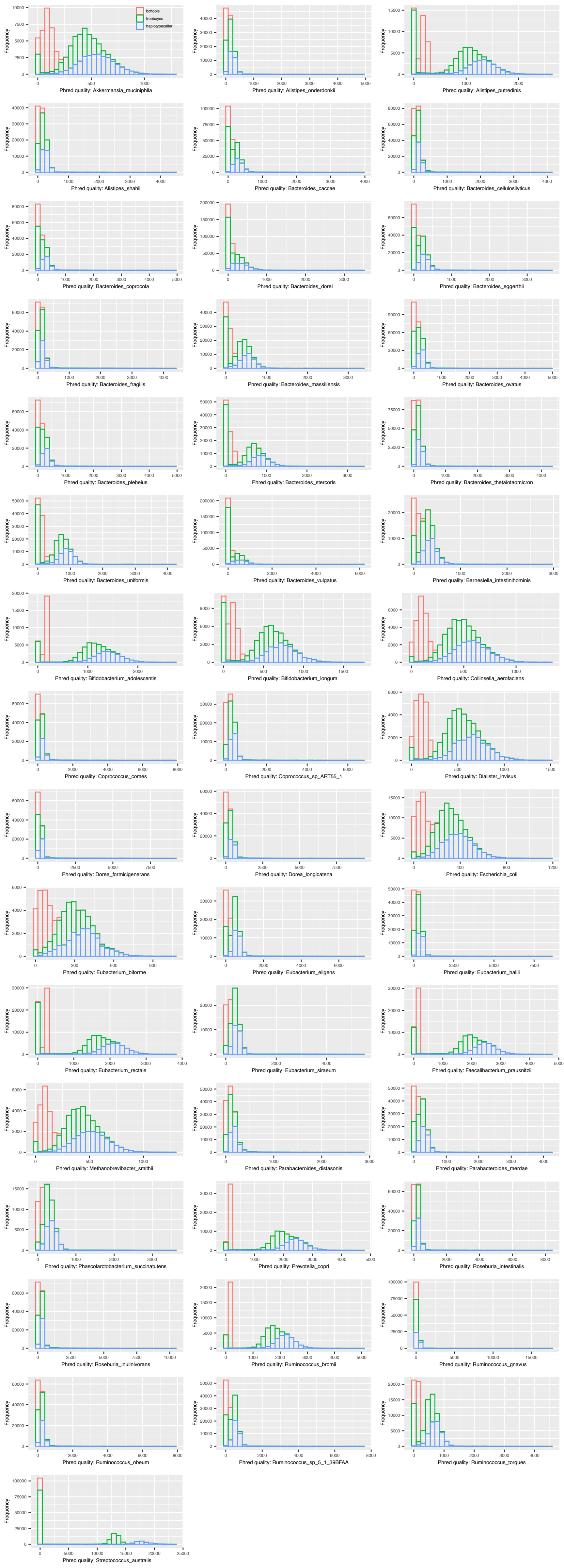

Supplement: Supplementary Figure 4 — Variant Phred quality distribution in each of the 46 analyzed species in BCFtools, HaplotypeCaller and Mutect2. [file Data_Sheet_4.PDF]

uniref

a

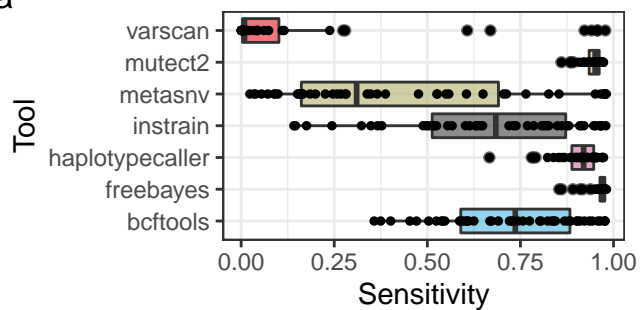

b

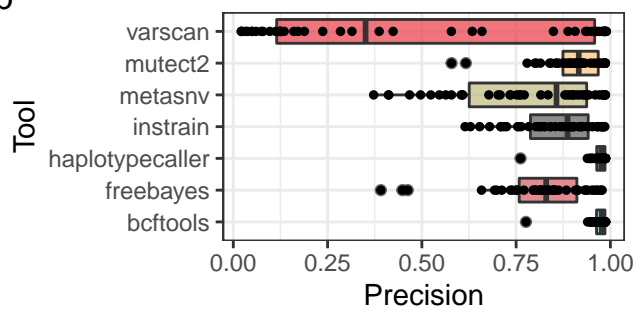

d

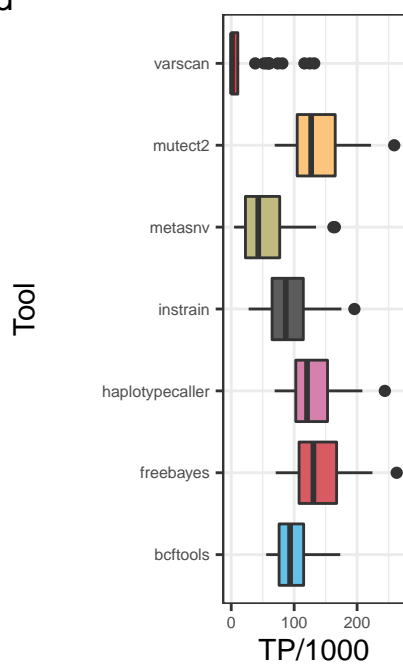

e

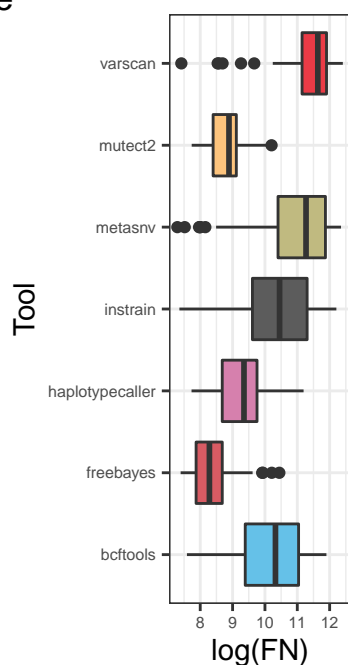

f

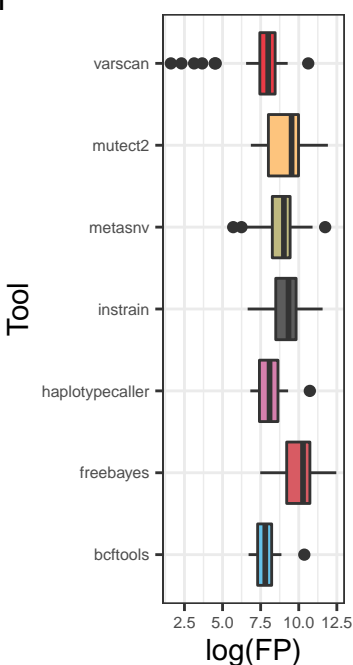

c

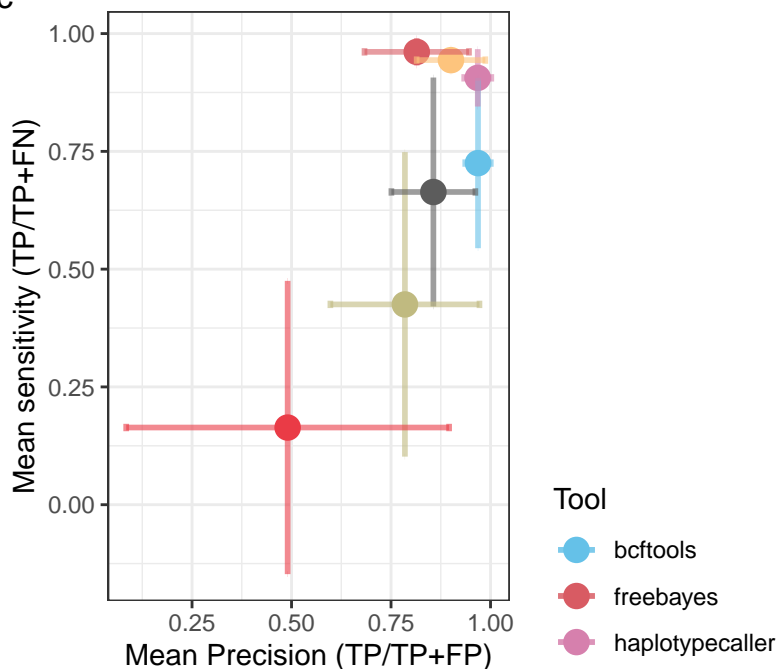

Supplement: Supplementary Figure 5 — Two-strain variant calling statistics of the seven tools. Colors indicated the tools. (A) Sensitivity (TP/TP + FN) of each tool. Tukey’s box plot shows the distribution of precision. Dots show precision per individual bacteria. (B) Precision (TP/TP + FP) of each tool. Tukey’s box plot shows the distribution of precision. Dots show precision per individual bacteria. (C) Precision vs. sensitivity plot. Dots present mean values among all bacteria. Error bars represent the standard deviation from the mean. Distribution of (D) TP, (E) FN, and (F). FP per tool, shown as Tukey’s box plots. Individual dots present bacteria >1.5 times the interquartile distance. [file Data_Sheet_5.PDF]
